# White Feces Syndrome of Shrimp Arises from Transformation, Sloughing and Aggregation of Hepatopancreatic Microvilli into Vermiform Bodies Superficially Resembling Gregarines

**DOI:** 10.1371/journal.pone.0099170

**Published:** 2014-06-09

**Authors:** Siriporn Sriurairatana, Visanu Boonyawiwat, Warachin Gangnonngiw, Chaowanee Laosutthipong, Jindanan Hiranchan, Timothy W. Flegel

**Affiliations:** 1 Center of Excellence for Shrimp Molecular Biology and Biotechnology (Centex Shrimp), Faculty of Science, Mahidol University, Bangkok, Thailand; 2 Department of Farm Resources and Production Medicine, Faculty of Veterinary Medicine, Kasetsart University, Thailand; 3 National Center for Genetic Engineering and Biotechnology, National Science and Technology Development Agency, Pratum Thani, Thailand; 4 Department of Biotechnology, Faculty of Science, Mahidol University, Bangkok, Thailand; Uppsala University, Sweden

## Abstract

Accompanying acute hepatopancreatic necrosis disease (AHPND) in cultivated Asian shrimp has been an increasing prevalence of vermiform, gregarine-like bodies within the shrimp hepatopancreas (HP) and midgut. In high quantity they result in white fecal strings and a phenomenon called white feces syndrome (WFS). Light microscopy (LM) of squash mounts and stained smears from fresh HP tissue revealed that the vermiform bodies are almost transparent with widths and diameters proportional to the HP tubule lumens in which they occur. Despite vermiform appearance, they show no cellular structure. At high magnification (LM with 40-100x objectives), they appear to consist of a thin, outer membrane enclosing a complex of thicker, inter-folded membranes. Transmission electron microscopy (TEM) revealed that the outer non-laminar membrane of the vermiform bodies bore no resemblance to a plasma membrane or to the outer layer of any known gregarine, other protozoan or metazoan. Sub-cellular organelles such as mitochondria, nuclei, endoplasmic reticulum and ribosomes were absent. The internal membranes had a tubular sub-structure and occasionally enclosed whole B-cells, sloughed from the HP tubule epithelium. These internal membranes were shown to arise from transformed microvilli that peeled away from HP tubule epithelial cells and then aggregated in the tubule lumen. Stripped of microvilli, the originating cells underwent lysis. By contrast, B-cells remained intact or were sloughed independently and whole from the tubule epithelium. When sometimes engulfed by the aggregated, transformed microvilli (ATM) they could be misinterpreted as cyst-like structures by light microscopy, contributing to gregarine-like appearance. The cause of ATM is currently unknown, but formation by loss of microvilli and subsequent cell lysis indicate that their formation is a pathological process. If sufficiently severe, they may retard shrimp growth and may predispose shrimp to opportunistic pathogens. Thus, the cause of ATM and their relationship (if any) to AHPND should be determined.

## Introduction

The results presented in this manuscript describing transformation, sloughing and aggregation of hepatopancreatic microvilli into vermiform bodies superficially resembling gregarines was obtained over a period of 6 years in a piecemeal fashion as a series of initially independent, sideline observations made during the course of dedicated research projects on a variety of known shrimp pathogens ranging from viruses to bacteria, fungi and parasites. It was not until very recently that the connections between the independent observations were understood, allowing them to be linked together into a coherent whole. A major activity that helped us to gain understanding of the connections between our piecemeal observations was the intensive research that has been carried out since 2009 and particularly since 2011 on many hundreds of shrimp specimens studied in attempts to determine the cause of acute hepatopancreatic necrosis disease (AHPND), the most recent, serious shrimp pandemic to cause severe losses in Asian shrimp cultivation.

AHPND Outbreaks began in cultivated shrimp *Penaeus (Penaeus) monodon* and *Penaeus (Litopenaeus) vannamei* China in 2009 and thereafter spread progressively to Vietnam (2010), Malaysia (2011) and Thailand (2012), although the cause of the disease was not known at that time. Indeed, it was not until 2011 that a case definition for AHPND was first described (referred to as acute hepatopancreatic necrosis syndrome or AHPNS at the time) by D.V. Lightner from the University of Arizona at a seminar organized by the Vietnamese Department of Animal Health in Hanoi in June 2011 (unpublished). It was subsequently described in the Global Aquaculture Advocate magazine under the heading of early mortality syndrome (EMS) in the Jan/Feb issue of 2012. Later in the same year, a disease card was prepared by the Network for Aquaculture Centres in Asia Pacific (NACA) and made available at its website (www.enaca.org). Finally, the causative agent (i.e., novel isolates of *Vibrio parahaemolyticus*) was discovered in 2013 [1]. The unique diagnostic characteristic of the disease is massive, medial sloughing of shrimp hepatopancreatic (HP) tubule epithelial cells as a result of a currently unknown toxin(s) that originates from the causative bacteria colonizing the shrimp stomach.

Since confirmatory diagnosis of AHPND is still dependent on histological diagnosis of massive sloughing of HP cells, many hundreds of cephalothorax tissue sections of shrimp suspected of AHPND infections have been examined with a primary focus on the shrimp hepatopancreas. During the course of this work on AHPND, a variety of other hepatopancreatic pathogens have also been encountered and recorded but are not often reported. Among such anomalies there has been an increasing prevalence of vermiform bodies that superficially resemble gregarines within the lumens of hepatopancreatic (HP) tubules, at the HP-stomach-midgut junction and in the midgut of cultivated giant tiger shrimp (*P. monodon*) and whiteleg shrimp (*P. vannamei*). They sometimes occur in sufficient quantities to cause white fecal strings in a phenomenon called white feces syndrome (WFS) in pond reared shrimp from approximately 2 months of culture onwards, and they were originally described as gregarines that caused WFS [2]. It has been estimated [3] that Thai production losses due to WFS in 2010 were 10–15% based on decreased survival and smaller harvest sizes of shrimp from WFS ponds, and this estimate excluded the normal annual Thai losses to white spot disease.

Here we describe detailed studies of the vermiform bodies using light and electron microscopes and show that they are not independent organisms but formations consisting of aggregated, transformed microvilli (ATM) that have originated by sloughing from epithelial cells of the shrimp hepatopancreatic tubules. They then accumulate at the HP-midgut junction before being discharged within feces via the midgut.

## Materials and Methods

As stated in the introduction, the work described in this manuscript was not the result of a dedicated study on ATM but constitutes the accumulation of piecemeal observations on many hundreds of shrimp samples obtained from many sources including 1) shrimp submitted to us by shrimp farmers for free diagnosis of a variety of diseases including EMS/AHPND, 2) shrimp purchased from farms or from broodstock producers for research or for laboratory training purposes and 3) shrimp donated by farmers or broodstock producers as a result of our requests at local presentations about what we now call ATM. In all cases, the specimens used came with no attached constraints in the publication of results obtained from their examination. Many of these shrimp specimens were of grossly normal appearance and showed normal histopathology (except perhaps for ATM). The vast majority of the histology slides examined since 2012 have been from shrimp ponds suspected of EMS/AHPNS outbreaks and none of these ponds (mostly at less than 35 days cultivation) have exhibited shrimp with gross signs of WFS because of the early stage of culture, but many show the presence of ATM. One passive surveillance of ponds exhibiting WFS outbreaks was carried out from 2009 to 2010 using 25 ponds at approximately 2 months of culture on 13 farms in the middle and eastern part of Thailand. In that survey, farmers collected approximately 10 shrimp from each pond, and transported them to the laboratory live for free examination for the presence of what we now call ATM by squash mount preparation.

The live shrimp obtained for various purposes and used piecemeal for analysis of ATM ranged from post-larvae to adult stages and were examined continually from 2009–2013. To prepare wet mounts, shrimp were first immersed in ice water for stunning and then surface sterilized with 70% ethanol before removal of small portions of HP tissue for direct observation in artificial sea water (25 ppt)(Marinium) by light microscopy or for preparing smears. For smears, a small drop of artificial seawater (25 ppt) or 2.7% NaCl solution was placed on a microscope slide. The HP tissue was placed in the solution before smearing. The smears were dried at 40°C before staining with H&E in the same manner as tissue sections (see below). For histological examination of tissue sections by light microscopy, shrimp were similarly stunned, injected with Davidson's fixative and processed for tissue sections of the cephalothorax stained with hematoxylin and eosin (H&E) by standard methods [4]. For electron microscopy, HP tissue of shrimp shown to carry ATM in whole mounts was cut in small portions of approximately 1 mm^3^, fixed in artificial sea water (Marinium) at 30 ppt containing 4% TEM grade glutaraldehyde for 24 hours before transfer to phosphate buffer (40 mM NaH_2_PO4·H_2_O, 100 mM Na_2_HPO4, 170 mM NaCl, pH 7.4). The pieces were then post-fixed in 1% OsO4 in the same buffer at 4°C for 1 h prior to being processed routinely for conventional embedding in Spurr's resin. Semithin sections for viewing by LM were stained with toluidine blue (toluidine blue O 4 g, pyronin 1 g, borax 5 g, distilled water 500 ml) and ultrathin sections for TEM were stained with uranyl acetate and lead citrate before viewing with a Hitachi H-500 transmission electron microscope.

## Results

### Field signs of white feces syndrome (WFS)

**Figure 1:**
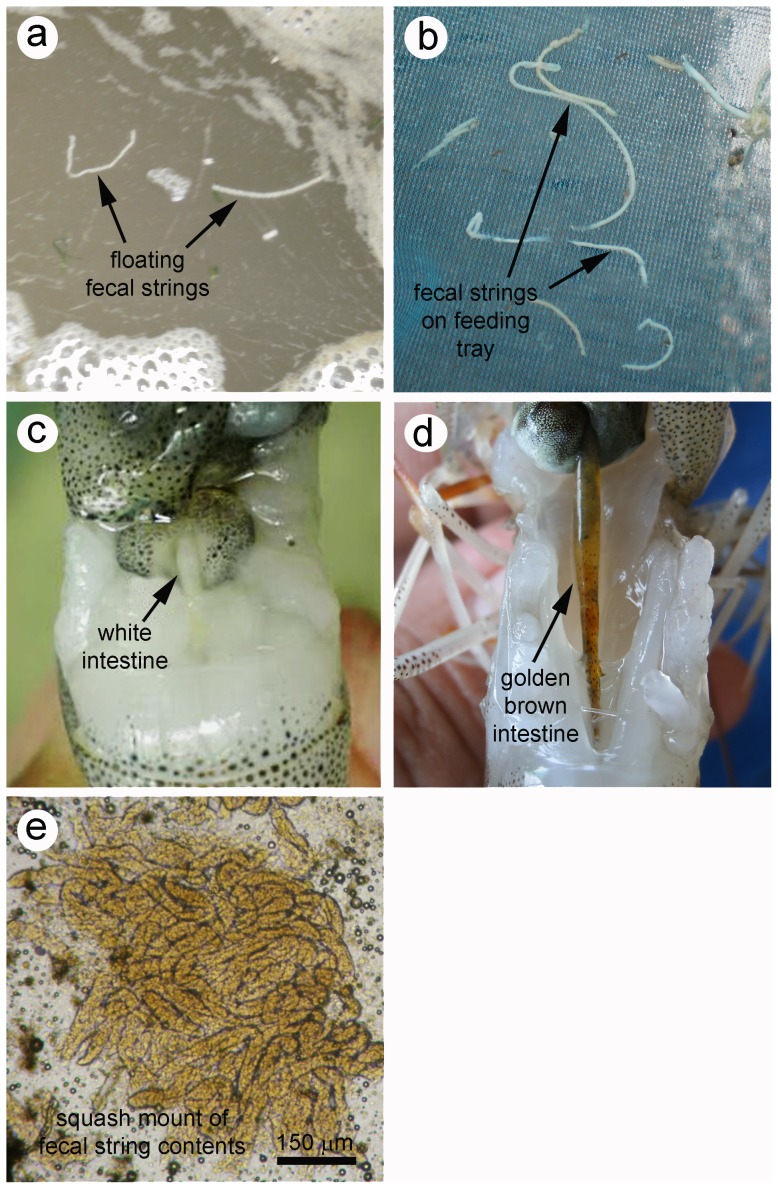
Gross signs of white feces syndrome WFS. (a) Floating, white fecal strings. (b) White fecal strings on a feeding tray. (c) White intestine of affected shrimp. (d) Golden brown intestine of an affected shrimp. (e) Photomicrograph of fecal string contents.

Gross signs of WFS in shrimp cultivation ponds (**Fig. 1**) included white to somewhat yellow, floating fecal strings (Fig. 1a) that sometimes collected in mats or could also be found on feeding trays (Fig. 1b). Examination of shrimp from ponds exhibiting signs of WFS revealed that the dissected midgut junction and midgut were distended and filled with white to yellow-golden contents (Fig. 1c,d). When the contents of the gut or fecal strings were examined in squash mounts with the light microscope, they consisted of masses of vermiform bodies that superficially resembled gregarines (Fig. 1e).

A passive surveillance of WFS outbreaks in *P. vannamei* was carried out from 2009 to 2010 in the eastern and middle part of Thailand in 25 ponds from 13 farms to determine the relationship between WFS and vermiform bodies resembling gregarines. The results revealed that 96% (24/25) of the ponds exhibiting WFS contained shrimp specimens that presented vermiform bodies resembling gregarines. Severely affected ponds exhibited reduction in shrimp survival by 20–30 percent when compared to normal ponds. There was also a decrease in feed consumption and growth rates were reduced as revealed by average daily weight gain (ADG) for the whole crop operation of less than 0.1 g/day compared to 0.2 g/day in normal ponds. Feed conversion ratios (FCR) ranged from 1.7 to 2.5 when compared to 1.5 or less for normal ponds.

### Light microscopy

**Figure 2:**
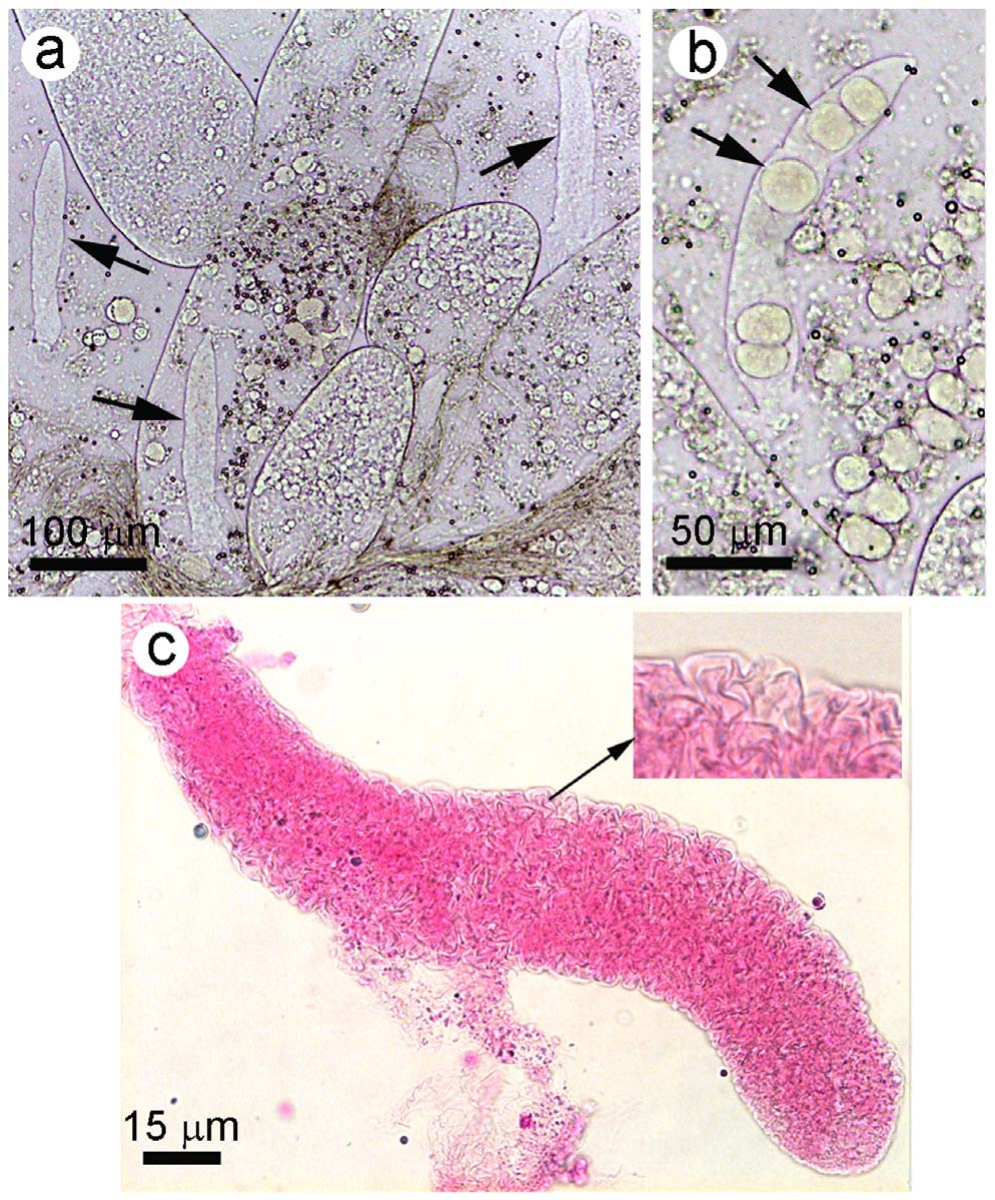
Squash mount of vermiform bodies (ATM) in shrimp hepatopancreatic tissue. (a) Low magnification photomicrograph showing 3 ATM with the central one inside an HP tubule. (b) Higher magnification photomicrograph showing an ATM containing cyst-like structures later found to be sloughed B-cells. (c) High magnification of an ATM stained with Rose Bengal to more clearly reveal its internal membranous structure.

**Figure 3:**
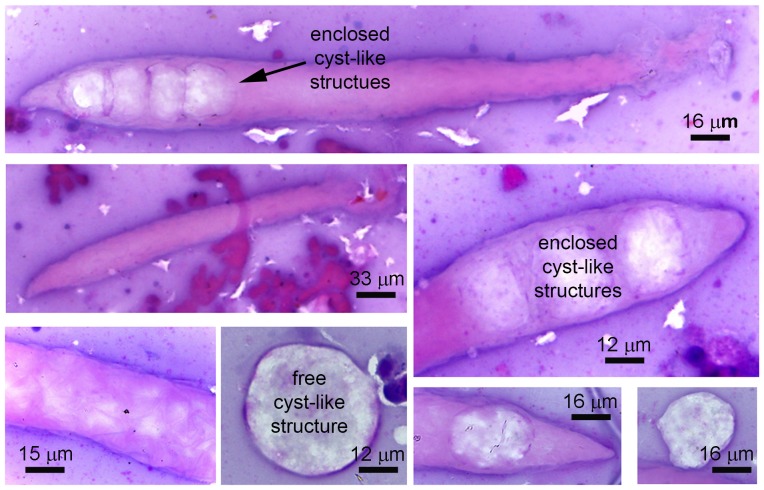
HP tissue smear stained with hematoxylin and eosin to reveal ATM. The photomicrographs clearly show the vermiform morphology and the cyst-like contents inside or free.

Whole mounts of shrimp hepatopancreatic tissue at any life stage from post-larvae (PL) to broodstock of *P. monodon* and *P. vannamei* currently cultivated in Asia (either diseased or grossly normal) often revealed the presence of non-motile, vermiform bodies superficially resembling gregarines within the tubule lumens, the HP-midgut junction and the midgut (**Fig. 2**). The average size was 39 (range 17 to 58) µm wide by 279 (50 to 517) µm long (N = 21) and was smaller but roughly proportional to the size of the tubules that contained them. They sometimes contained spherical bodies that resembled cysts (Fig. 2b). Besides these cyst-like inclusions, they had no apparent cellular or subcellular features (e.g., nuclei) and contained only what appeared to be interfolded membranes that could be visibly enhanced by staining with Rose Bengal (Fig. 2c). Smears of HP tissue from affected shrimp stained with hematoxylin and eosin made these bodies more clearly visible but still revealed no cellular or subcellular structures such as nuclei (**Fig. 3**). However, examination of these two types of preparations clearly revealed why they were first referred to as gregarine-like entities (GLE).

Examination of living shrimp specimens of both *P. monodon* and *P. vannamei* from post larval to broodstock life stages often revealed the presence of GLE in small to massive numbers that varied from shrimp to shrimp specimen, even from the same pond or hatchery tank. The affected shrimp showed no gross signs of disease (including white fecal strings) resulting from their presence, even in high numbers.

**Figure 4:**
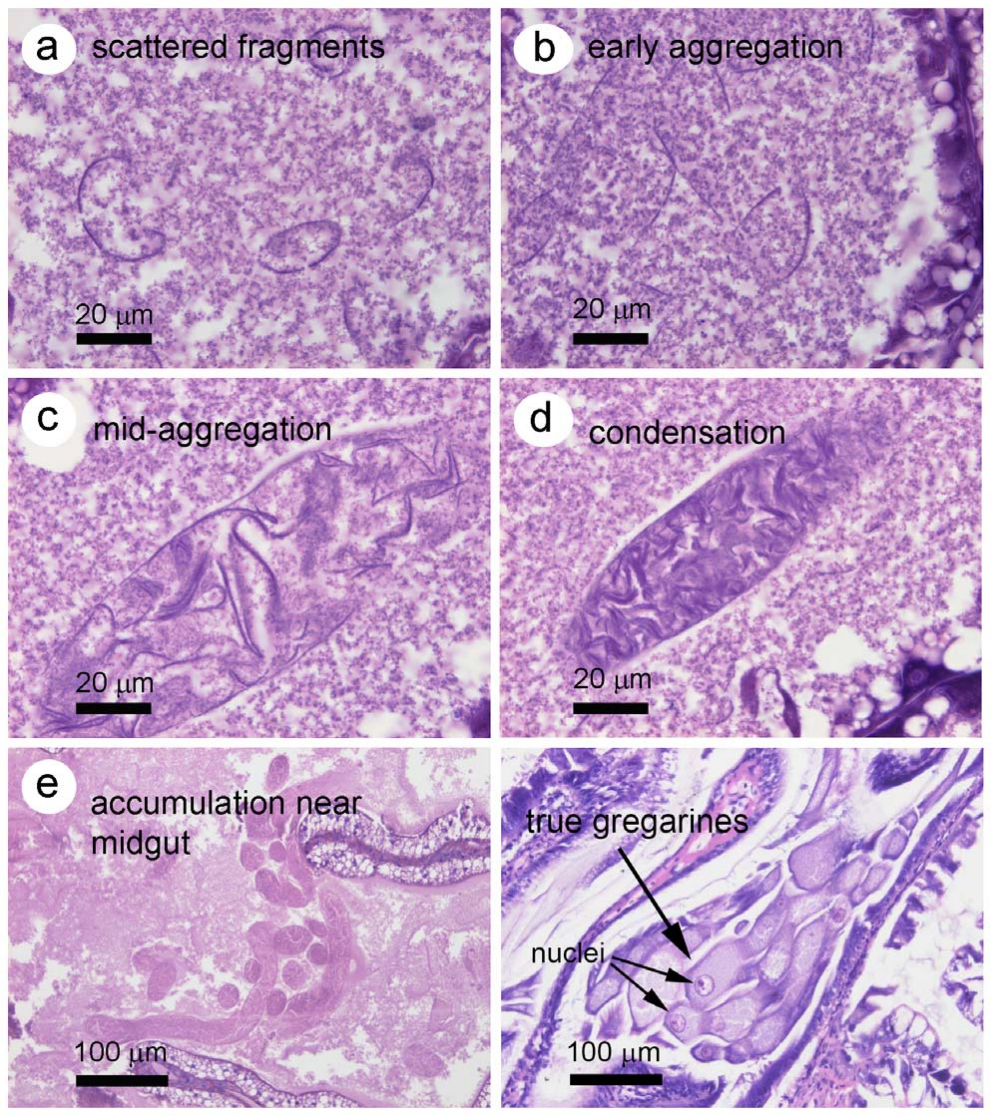
ATM aggregation steps in H&E stained HP tissue sections in comparison to true gregarines. (a) Small, scattered membrane-lie structures in the HP tubule lumen. (b) More extended membranes beginning to aggregate in the tubule lumen. (c) Tighter aggregation of membranes bound by a continuous outer membrane and taking the shape of ATM. (d) Highly condensed ATM in a tubule lumen. (e) Accumulation of many individual ATM at the center of the HP near the midgut junction. (f) True gregarines clustered near the midgut junction and showing prominent nuclei.

HP tissue of affected shrimp fixed with Davidson's fixative and processed for normal histological examination of tissue sections stained with hematoxylin and eosin (H&E) did not give as good resolution of the GLE as did whole squash mounts or stained smears. Instead, they often appeared to be partially degraded by the preparation steps, so that their membrane contents were difficult to resolve or could not be distinguished easily from the normal chyme-like material that is often seen in the HP lumens of shrimp that are actively feeding. In some better preserved specimens, it was possible to prepare a series of photomicrographs suggestive of progressive aggregation and condensation of individual membranes into GLE that lacked recognizable cellular structures and accumulated at the center of the HP near the midgut junction to superficially resemble gregarines (**Fig. 4**). The progression began as scattered membranes (**Fig. 4a**) followed by stages of aggregation (**Figs.** **4b & 4c**) followed by condensation (Fig. **4d**) and accumulation at the HP center (**Fig. 4e**). For comparison, a photomicrograph of H&E stained tissue of cultivated *P. monodon* (**Fig. 4f**) shows true gregarine trophozooites (probably *Nematopsis* sp. [5])clustered in the region of the HP/midgut junction. These are rarely found in cultivated shrimp in Thailand and compared to GLE, they are larger, stain more intensely and have prominent nuclei. In addition, they do not arise by a process of membrane aggregation and condensation.

**Figure 5:**
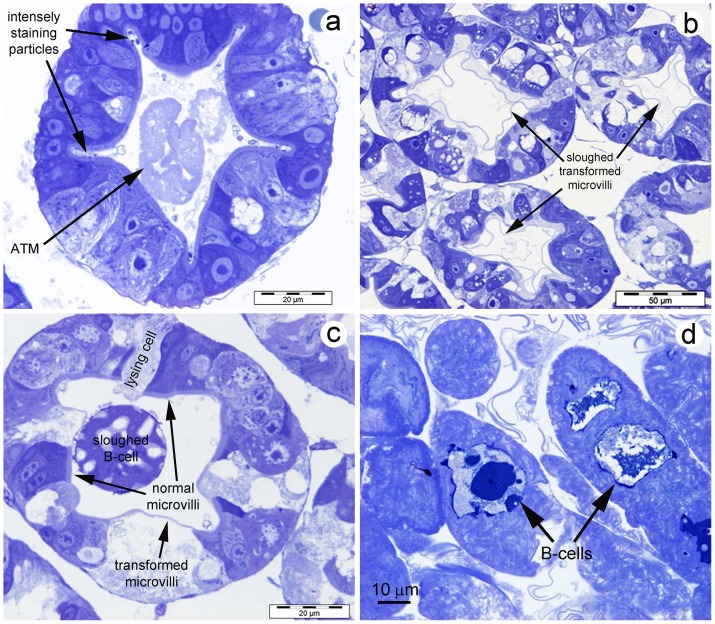
Semi-thin sections of HP tissue stained with toluidine blue. (a) Cross section of an HP tubule near the distal end showing densely stained particles in crypts formed by folds of the tubule epihelium and showing aggregated, transformed microvilli (ATM) in the tubule lumen. Note that microvillar layers of all the cells are intact. (b) Cross sections of HP tubules showing sloughed, transformed microvilli. (c) Cross section of an HP tubule showing a modified, sloughed B-cell in the tubule lumen with microvilli scattered over its surface. Also seen are tubule epithelial cells with normal microvilli and transformed mivrovilli, and one cell denuded of microvilli, undergoing lysis. (d) High magnification of clustered ATM at the center of the HP clearly showing an outer membrane enclosing multitudes of folded transformed microvilli. Some also contain enclosed, sloughed B-cells. Note many free transformed microvilli fragments surrounding the ATM.

Using light microscopy to examine semi-thin sections of HP tissue of affected shrimp fixed and embedded for transmission electron microscopy (TEM) and stained with toluidine blue, clearly revealed that the GLE consisted of a thin outer membrane that enclosed a complex of thicker interfolded membranes (**Fig. 5a–d**). These sometimes surrounded what was later found to be sloughed, whole B-cells (**Fig. 5d**). The tubules that contained the GLE also showed many epithelial cells with abnormally thin microvillar layers or denuded of microvilli (**Fig. 5c** **)**. The latter showed signs of lysis (**Fig. 5c**). Free membrane-like structures were present in the tubule lumens in addition to the GRL (**Fig. 5b,d**).

### Transmission electron microscopy (TEM)

**Figure 6:**
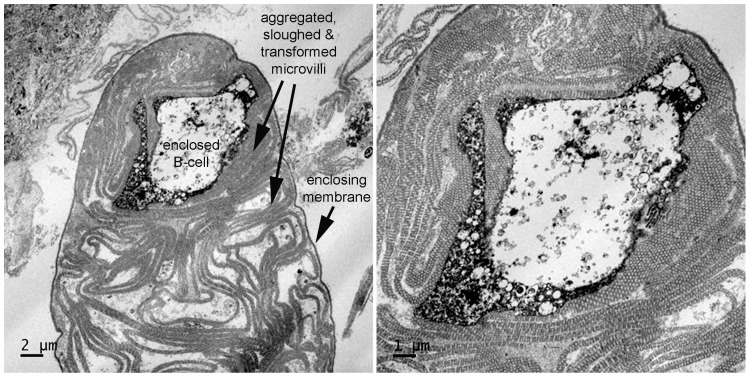
TEM of an ATM structure containing an enclosed, sloughed and modified B-cell. Note the single-layer enclosing membrane and the internal complex of sloughed, modified microvilli.

**Figure 7:**
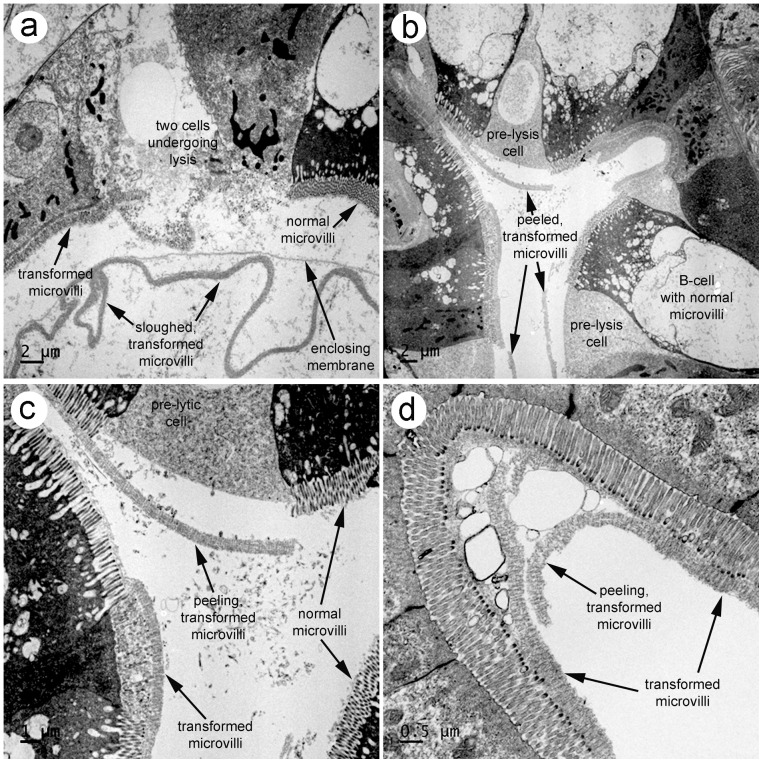
TEM of steps in microvillar transformation and sloughing. (a) Low magnification of HP tubule epithelial cells showing normal and transformed microvilli and two denuded cells undergoing lysis. Also shown is an early stage in the aggregation of transformed and sloughed microvilli surrounded by an enclosing membrane. (b) Low magnification of HP tubule epithelial cells with transformed microvilli peeling from the cell surface, prior to cell lysis. (c) Higher magnification of the field from (b) clearly showing the difference between normal and transformed microvilli. (d) High magnification of HP tubule epithelial cells showing the tubular nature of transformed and peeled microvillar layers.

TEM of affected shrimp tissues (**Fig. 6**) revealed that the GLE were surrounded by a thin, single-layer membrane that bore no resemblance to a bilaminar plasma membrane or to the complex outer layers of known protozoans, metazoans or gregarines (http://tolweb.org/Gregarina/124806; [6], [7], [8], [9], [10], [11], [12], including a gregarine previously reported from Thailand [5]. It enclosed a complex of thicker, interfolded membranes with a tubular substructure, and these occasionally surrounded whole B-cells sloughed from the HP tubule epithelium. The GLE otherwise contained no recognizable cellular contents such as nuclei, mitochondria, ribosomes, etc. The origin of the outer, single-layer membrane could not be determined, but the enclosed, interfolded membranes with a tubular substructure were found to originate from microvilli of HP tubule epithelial cells of the R and F types (**Fig. 7**). The microvilli first became transformed into a partially collapsed state (**Fig.** **7a,c,d**) before they peeled off of the cells (**Fig. 7b,c,d**) and sloughed into the tubule lumen where they aggregated to form GLE (**Fig. 7a**). The cells denuded of microvilli subsequently lysed (**Fig. 7a**), releasing their contents into the tubule lumen, often leaving a remnant containing the basal nucleus collapsed against the tubule basal membrane. Based on all the information from light microscopy to electron microscopy, these bodies were named aggregated transformed microvilli (ATM).

**Figure 8:**
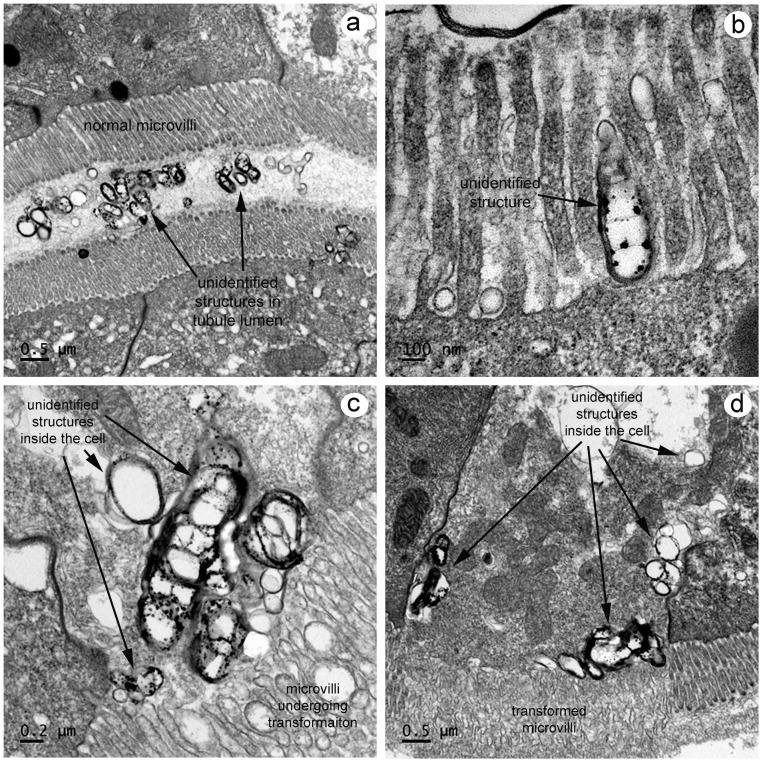
TEM of unusual electron-dense particles in HP tubule crypts. (a) Low magnification of electron-dense particles of highly variable shape in the HP tubule lumen between layers of normal microvilli from facing epithelial cells. (b) High magnification of one of the electron-dense particles between the microvilli on the outside surface of an epithelial cell, possibly prior to cell entry. (c) High magnification of electron dense particles inside an epithelial cell with adjacent microvilli on the cell surface undergoing morphological changes. (d) Low magnification of an epithelial cell containing large numbers of electron dense particles and with microvilli in an advanced stage of transformation.

By TEM, the F and R cells with transformed microvilli did not show the presence of recognizable pathogens such as viruses, bacteria or parasites. However, in the E-cell region of the HP where the tubule epithelium was somewhat folded to form cript-like spaces with facing microvillar layers, minute, densely staining bodies of irregular shape and size could be seen by light microscopy (Fig. 4a) and by TEM (**Fig. 8**). These appeared to aggregate and be capable of passing through the microvillar layers (**Fig. 8a & b**) to enter the subtending cellular cytoplasm (**Fig. 8c**). This association appeared to be accompanied by changes in the morphology of the microvilli (**Fig. 8c & d**), and cells that showed advanced microvillar transformation appeared to have large numbers of such inclusions (**Fig. 8d**). It was not clear whether they had increased in numbers by accumulation or by proliferation. They lacked subcellular structures that might indicate relationship to known viral, prokaryotic or eukaryotic organisms, and it could not be determined whether they were causal or incidental to ATM formation.

We examined the shrimp midgut only in squash mounts and in the portion of the midgut that occasionally appeared in saggital tissue sections of the whole cephalothorax region in H&E stained slides. We did not examine the midgut further with semithin sections or by TEM. However, in the H&E sections we found no evidence that ATM were formed in the midgut. Instead, they simply accumulated there as they were released from the HP.

## Discussion

Before we had detailed results from electron microscopy revealing the nature of ATM origin, we were dependent on light microscopy results showing vermiform bodies that resembled gregarines. A search of the literature about gregarines at that time revealed that none of those previously reported from shrimp [13], [14], [15], including one previously reported from Thailand [5] bore any resemblance to ATM. Specifically, they lacked motility (i.e., in squash mounts) and showed no organelles such as usually prominent nuclei. Subsequent results from electron microscopy confirmed the absence of nuclei and other ultrastructural features normally found in gregarines (e.g., complex pellicles, mitochondria, ribosomes, etc.). However, we did find a previous report by Johnson [16] that described the occasional presence of cellophane-like aggregations of membranous material in the hepatopancreatic tubule lumens of crabs and proposed that they arose from the microvilli of the tubule epithelial cells. She did not give details of their formation and expressed the inability to explain their function or significance. She also expressed the inability to conclude whether they were the result of a normal process or of some kind of rare pathology. Our ATM from shrimp morphologically resembled the structures described by Johnson in crabs by light microscopy (her Figs. 129, 147 and 164) and especially by TEM (her Fig. 152). We could find no other published description of these entities in crustaceans.

When the occurrence of ATM is severe, it can lead to the formation of white fecal strings in shrimp, and if many shrimp in the same pond exhibit this phenomenon, it can lead to floating fecal strings that sometimes accumulate in floating mats (i.e., white feces syndrome or WFS). This usually occurs from 2 months of cultivation onwards, and it may be accompanied by high shrimp mortality. However, ATM sometimes occur together with shrimp hepatopancreatic diseases such as the AHPND, other types of vibriosis, and parasitemia with the microsporidian *Enterocytozoon hepatopenaei*. As a result, the cause of WFS in Vietnam was attributed also to *E. hepatopenaei* [17], but this was later shown to be very unlikely based on closer study of natural and laboratory infections of *E. hepatopenaei* in Thailand [18]. Thus, it is certain that these severe cases of WFS result from massive ATM formation. However, the cause of ATM formation remains a mystery. Hopefully, understanding their nature and mode of formation will lead to more directed studies to determine their cause.

Overall, our work has shown that the formation of ATM results from transformation of microvilli followed by sloughing from the subtending cell and by subsequent lysis of that cell. These features indicate that ATM formation is a pathological process. The fact that ATM were not previously described in shrimp, despite their easy recognition in whole mounts and smears of HP tissue from living shrimp, argues that their previous occurrence was probably overlooked due to low prevalence, as previously reported by Johnson for crabs [16]. However, they have recently increased in prevalence in Asia to the extent that they are now too prevalent to be overlooked.

It may be significant that the increase in prevalence of ATM has been coincidental with the increase in prevalence of AHPND outbreaks. Although this might suggest a possible causal association, there has also been a coincidental increase in prevalence of the hepatopancreatic microsporidian *Enterocytozoon hepatopenaei* [18] with AHPND, and we now know that this is certainly not a causal relationship, since AHPND is caused by unrelated bacteria [1]. So at least for *E. hepatopenaei* and AHPND bacteria, it seems likely that some of their increased prevalence has resulted from contamination of broostock and/or post-larvae (PL). This contention is supported by anecdotal evidence from widely separated Thai shrimp farmers who received portions of single batches of PL derived from SPF shrimp stocks but then experienced AHPND outbreaks more-or-less simultaneously. It is also supported by our discovery of endemic *E. hepatopenaei* in locally held broodstock and PL of SPF *P. vannamei* stocks that originated from the Americas where this microsporidian has never previously been reported [18].

Altogether the previous information suggests that the biosecurity measures in at least some shrimp hatcheries have not been sufficiently rigorous to exclude contamination by imported and/or local pathogens. Therefore, we must consider two possibilities with respect to ATM. Either they are caused by a new agent that has contaminated SPF stocks in a similar manner to AHPND and *E. hepatopenaei*, or that they constitute an alternate manifestation of an existing pathogen. For example, it has been reported that AHPND bacteria produce a potent toxin that can cause sloughing of hepatopancreatic tubule epithelial cells [1], and it may be asked whether the same toxin at low doses may cause the formation of ATM in the absence of cell sloughing. To test this latter possibility, the laboratory infection model recently described [1] may be used with diluted toxin preparations from the causative bacteria. With respect to the existence of a new pathogen, comparative metagenomic analysis of shrimp with and without ATM may be the most useful. Alternatively, the possible involvement of the minute, electron dense bodies described here to be associated with microvillar transformation may be further investigated. For example, it may be possible to separate them physically from tissue homogenates by differential centrifugation and/or filtration for further analysis and for laboratory challenge tests.

In conclusion, we have revealed by TEM that vermiform structures superficially resembling gregarines and commonly found now in the HP of Asian cultivated shrimp are not independent organisms but result from the transformation, sloughing and aggregation of microvilli from the HP tubule epithelial cells themselves. The denuded epithelial cells subsequently undergo lysis, indicating that the process has the potential for negative impact on shrimp growth and survival, and in very severe cases can lead to the phenomenon called white feces syndrome (WFS). Further investigation is needed to understand the cause of ATM and evaluate their impact on shrimp production.
